# A unified classification approach rating clinical utility of protein biomarkers across neurologic diseases

**DOI:** 10.1016/j.ebiom.2023.104456

**Published:** 2023-02-04

**Authors:** Alexander M. Bernhardt, Steffen Tiedt, Daniel Teupser, Martin Dichgans, Bernhard Meyer, Jens Gempt, Peer-Hendrik Kuhn, Mikael Simons, Carla Palleis, Endy Weidinger, Georg Nübling, Lesca Holdt, Lisa Hönikl, Christiane Gasperi, Pieter Giesbertz, Stephan A. Müller, Stephan Breimann, Stefan F. Lichtenthaler, Bernhard Kuster, Matthias Mann, Axel Imhof, Teresa Barth, Stefanie M. Hauck, Henrik Zetterberg, Markus Otto, Wilko Weichert, Bernhard Hemmer, Johannes Levin

**Affiliations:** a Department of Neurology, Ludwig-Maximilians-Universität München, Munich, Germany; b German Center for Neurodegenerative Diseases, Site Munich, Germany; c Munich Cluster for Systems Neurology (SyNergy), Munich, Germany; d Institute for Stroke and Dementia Research, University Hospital, LMU Munich, Munich, Germany; e Institute of Laboratory Medicine, University Hospital, LMU Munich, Munich, Germany; f Department of Neurosurgery, Klinikum Rechts der Isar, School of Medicine, Technical University of Munich, Munich, Germany; g Institute of Pathology, Technische Universität München, Munich, Germany; h Institute of Neuronal Cell Biology, Technical University Munich, 80802, Munich, Germany; i Department of Neurology, Klinikum Rechts der Isar, Technical University of Munich, Munich, Germany; j Neuroproteomics, School of Medicine, Klinikum Rechts der Isar, Technical University of Munich, Munich, Germany; k Department of Bioinformatics, Wissenschaftszentrum Weihenstephan, Technical University of Munich, Freising, Germany; l Chair of Proteomics and Bioanalytics, Technical University of Munich, Freising, Germany; m German Cancer Consortium (DKTK), Munich Partner Site, Munich, Germany; n German Cancer Research Center (DKFZ), Heidelberg, Germany; o Novo Nordisk Foundation Center for Protein Research, Faculty of Health and Medical Sciences, University of Copenhagen, Copenhagen, Denmark; p Department of Proteomics and Signal Transduction, Max Planck Institute of Biochemistry, Martinsried, Germany; q Protein Analysis Unit, Biomedical Center (BMC), Faculty of Medicine, Ludwig-Maximilians-University (LMU) Munich, Großhaderner Straße 9, 82152, Martinsried, Germany; r Research Unit Protein Science and Metabolomics and Proteomics Core, Helmholtz Centre Munich, German Research Center for Environmental Health, 85764, Neuherberg, Germany; s Department of Psychiatry and Neurochemistry, Institute of Neuroscience and Physiology, The Sahlgrenska Academy at the University of Gothenburg, Mölndal, Sweden; t Clinical Neurochemistry Laboratory, Sahlgrenska University Hospital, Mölndal, Sweden; u Department of Neurodegenerative Disease, UCL Institute of Neurology, Queen Square, London, UK; v UK Dementia Research Institute at UCL, London, UK; w Hong Kong Center for Neurodegenerative Diseases, Clear Water Bay, Hong Kong, China; x Department of Neurology, Halle University Hospital, Martin Luther University Halle/Wittenberg, Saale, Germany

**Keywords:** Biomarker, Neurology, Proteomics, Protein, Clinical utility, Analytical validity

## Abstract

A major evolution from purely clinical diagnoses to biomarker supported clinical diagnosing has been occurring over the past years in neurology. High-throughput methods, such as next-generation sequencing and mass spectrometry-based proteomics along with improved neuroimaging methods, are accelerating this development. This calls for a consensus framework that is broadly applicable and provides a spot-on overview of the clinical validity of novel biomarkers. We propose a harmonized terminology and a uniform concept that stratifies biomarkers according to clinical context of use and evidence levels, adapted from existing frameworks in oncology with a strong focus on (epi)genetic markers and treatment context. We demonstrate that this framework allows for a consistent assessment of clinical validity across disease entities and that sufficient evidence for many clinical applications of protein biomarkers is lacking. Our framework may help to identify promising biomarker candidates and classify their applications by clinical context, aiming for routine clinical use of (protein) biomarkers in neurology.

## Background

Neurologic diseases are the major cause of loss in disability-adjusted life-years.^1^ It is indispensable to elucidate underlying molecular mechanisms that allow an early molecular-biological diagnosis and a reliable stratification of disease subtypes as a basis for future targeted disease-modifying therapies. An increasing number of current diagnostic criteria, such as those for Alzheimer's disease (AD)^2^ and multiple sclerosis (MS),^3^ emphasize the importance of diagnostic molecular biomarkers (for a glossary of terms, see Panel 1). Genomic biomarker approaches have been of limited value in transforming prevention, diagnosis, and therapy not only of the most common polygenic, multifactorial neurodegenerative, neurovascular and neuroinflammatory, but also neurooncologic diseases. Most gliomas are caused by genomic alterations that cannot be exploited for targeted therapies directly. Within the vast landscape of molecular biomarkers, proteins—given their role as direct effectors of most biological processes—can provide a comprehensive picture of disease phenotypes beyond risk assessment using genetic information. Technological breakthroughs, such as targeted proteomics via mass spectrometry,^4^ high-throughput multiplex proteomic immunoassay panels (e.g., proximity extension assays^5^), single molecule arrays (Simoa)^6^ and improved automated immunoassays,^7^ open up new opportunities for clinical use. The goal of this review is to introduce a unified classification approach that can be broadly applied to protein biomarkers and covers all potential contexts of use. The potential clinical utility of selected protein biomarkers will be highlighted for neurodegenerative, neurovascular, neuroinflammatory and neurooncologic diseases.

Panel 1 **Glossary of terms.**

• **Analytical validation:** Analytic method validation is the process of determining of how accurately and reliably the biomarker of interest is measured in the patient tissue (in terms of its sensitivity, specificity, accuracy, precision, and other relevant performance characteristics using a specified technical protocol). This is validation of the test's, tool's, or instrument's technical performance, but is not validation of the item's usefulness.
• **Biological diagnosis:** A diagnosis based on only biomarker evidence.
• **Biological rationale:** (Patho-)physiological process the biomarker is associated with.
• **Biomarker:** a characteristic that is objectively measured and evaluated as an indicator of normal biological processes, pathogenic processes, or pharmacological responses to therapeutic intervention. Molecular, histologic, radiographic, or physiologic characteristics are types of biomarkers. Biomarkers are developed and validated through the process of analytical validation, clinical validation, and the demonstration of clinical utility.
• **Clinical endpoint:** A precisely defined and measurable variable intended to reflect an outcome of interest that is statistically analysed to address a particular research question. A precise definition of an endpoint typically specifies the type of assessments made, the timing of those assessments, the assessment tools used, and possibly other details, as applicable, such as how multiple assessments within an individual are to be combined.
• **Clinical outcome:** a measurable characteristic that describes or reflects how an individual feels, functions or survives.
• **Clinical qualification:** A conclusion, based on a formal regulatory process, that within the stated context of use, a biomarker can be relied upon to have a specific interpretation regarding the clinical endpoint.
• **Clinical utility:** actual usefulness/added value of the biomarker measurement in clinical routine considering the defined context of use.
• **Clinical validation:** Evaluating how robustly and reliably the biomarker measurement correlates with the clinical endpoint of interest.
• **Clinico–biological diagnosis:** A diagnosis based on both clinical and biomarker findings.
• **Context of use:** A statement that fully describes the context in which the biomarker measurement is to be used (within one of the broad seven BEST biomarker categories)
• **Cross-sectional biomarker study:** The biomarker is measured once in a cohort, case–control or case series at a specific point of time. This includes studies with longitudinal data collection as long as the biomarker of interest is analyzed at only one point in time per individual. This study type mandates de novo collection of data with a pre-specified study protocol comprising the biomarker of interest.
• **Longitudinal cohort biomarker study:** The biomarker is measured at least twice within a pre-defined time window. An appropriately characterized longitudinal cohort composed of patients with/at risk of the condition of interest, healthy controls and/or patients with other conditions (e.g., differential diagnoses to the condition of interest) is chosen. This study type mandates de novo collection of data with a pre-specified study protocol comprising the biomarker of interest.
• **Retrospective biomarker study:** All studies based on biomarker measurements and/or data collection that were already conducted. This category comprises retrospective chart reviews as well as ex post biomarker measurements conducted on samples/data from cross-sectional or longitudinal cohort biomarker studies that did not include the biomarker of interest in their original protocol.
• **Robustness:** A statistical test is defined as “robust” if the α risk (the probability of rejecting the null hypothesis—the hypothesis of no difference or effect when it is true) has little variation when the conditions for applying the test are not fully met. This definition is also applicable to biochemical tests and may be of value in test selection. For a test to be robust, the total pre-analytical and analytical error must be substantially lower than the percent fold change observed in the condition to be detected.
• **Surrogate endpoint:** An endpoint that is used as a substitute for a direct measure of how a patient feels, functions, or survives. A surrogate endpoint does not measure the clinical benefit of primary interest itself, but rather is expected to predict clinical benefit or harm based on epidemiologic, therapeutic, pathophysiologic, or other scientific evidence.

## Starting point: classification systems developed in oncology

Oncology pioneered concepts for classifying genomic molecular biomarkers with emphasis on their therapeutic relevance. As a starting point for our unified concept, we review the most widely used classification systems of genomic biomarkers in oncology, which focus on gene variant interpretation, namely:

• Joint consensus recommendation (JCR) for the interpretation and reporting of sequence variants in cancer by the Association for Molecular Pathology (AMP), American College of Medical Genetics and Genomics (ACMG), American Society of Clinical Oncology (ASCO) and College of American Pathologists (CAP)^8^
• ESMO scale for clinical actionability of molecular targets (ESCAT)^9^
• National center for tumour diseases classification (NCT)^10^
• CIViC (Clinical Interpretation of Variants in Cancer)^11^

Supplementary Fig. S1 provides an overview based on a recently published comparison of these classification systems.^12^ It is important to note that these are specifically tailored to genetic biomarkers and their clinical utility with regard to targeted therapies.

JCR, for example, employs a four-tiered classification system to rate therapeutic relevance of genomic alterations. It is based on preclinical and clinical evidence as well as regulatory approval status by the FDA. Tier 1 comprises variants of strong clinical significance: biomarkers that predict response or resistance to FDA-approved therapies for a specific type of tumour, biomarkers included in professional guidelines as well as biomarkers based on well-powered studies with consensus from experts in the field. In contrast, tier 2 covers variants of potential clinical significance: biomarkers that predict response or resistance to FDA-approved therapies for a different type of tumour or biomarkers of the same type of tumour where only preclinical data are available. Variants of unknown clinical significance (no convincing evidence of cancer association) are classified as tier 3, whereas tier 4 denotes benign or likely benign variants (no existing evidence of cancer association).

In addition to the aforementioned classification systems that combine evidence levels with therapeutic relevance, we consider a five-phase framework for the development of cancer-screening biomarkers^13^ adopted by the Early Detection Research Network (EDRN) that is particularly relevant to rate risk and diagnostic biomarkers. This framework was proposed as a roadmap for the development of biomarkers associated with other diseases, such as Alzheimer's disease.^14^ The EDRN roadmap defines five phases of biomarker development encompassing 1) preclinical exploratory assay development 2) clinical assay validation (estimate the frequency of true-positive and false-positive results or ROC curves), 3) retrospective longitudinal studies 4) prospective diagnostic accuracy (calculating frequencies of positive and false-positive detection) studies and 5) disease burden reduction studies (estimating reductions in mortality, morbidity, and disability associated with biomarker testing). Uniquely, this roadmap combines issues of both analytical and clinical validity (see Panel 1).

## A unifying classification concept

**Fig. 1:**
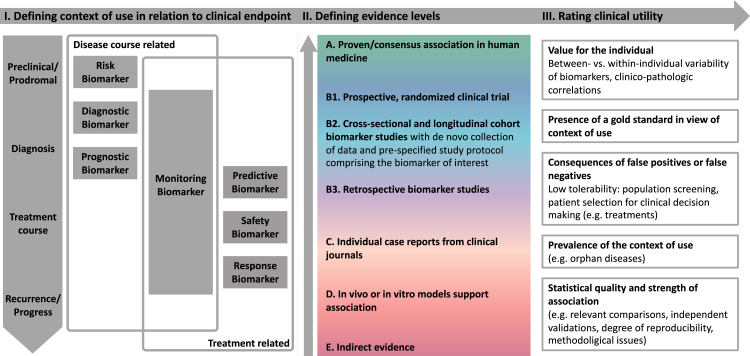
**Unifying classification concept for protein biomarkers across neurologic disease entities.** According to the unified classification, a biomarker is first classified by its clinical application by grouping it into one of the seven categories and by precisely defining the associated clinical endpoint. In a second step, the available evidence is summarized in one of the levels A–E. Rating clinical utility in a third step is difficult to operationalise. Depending on the individual patient case and disease, the one or other approach may be more suitable. It is important to note whether a gold-standard for the measurement of the clinical endpoint already exists or whether there is a general lack of appropriate biomarkers.

Our approach is based on an application-oriented taxonomy employed by both the FDA and EMA in their biomarker qualification programs (Panel 1, Panel 2).^15^^,^^16^ Biomarkers can be categorized into seven groups as suggested by a FDA/NIH biomarker working group^17^ that cover the entire clinical continuum of a disease (Fig. 1. I, Panel 2). Susceptibility/risk, diagnostic, prognostic, and monitoring biomarkers are employed for measuring clinical endpoints delineating the course of a disease, whereas predictive, pharmacodynamic/response and safety biomarkers depict treatment related clinical endpoints. Monitoring biomarkers are both disease course and treatment related.

Panel 2 **Categories of clinical applications of biomarkers.**

• **Susceptibility/risk biomarker:** A biomarker that indicates an increased potential to develop a disease or medical condition in an individual who does not currently have clinically apparent disease or the medical condition.
• **Diagnostic biomarker:** A biomarker used to either detect/confirm the presence of a disease or condition of interest or to characterize certain aspects of the disease.
• **Prognostic biomarker:** A biomarker used to identify the likelihood of a clinical event, spontaneous remission, disease recurrence or (rate of) progression in patients who have the disease or medical condition of interest.
• **Monitoring biomarker:** A biomarker measured repeatedly to assess the status of a disease or medical condition or to quantify exposure to a medical product or an environmental agent.
• **Predictive biomarker:** A biomarker used to identify individuals who are more likely than similar individuals without the biomarker to experience a favorable or unfavorable effect from exposure to a medical product or an environmental agent. Unlike prognostic biomarkers, predictive biomarkers are linked to treatment.
• **Pharmacodynamic/response biomarker:** A biomarker used to show that a biological response has occurred in an individual who has been exposed to a medical product or an environmental agent.
• **Safety biomarker:** A biomarker measured before or after an exposure to a medical product or an environmental agent to indicate the likelihood, presence, or extent of toxicity as an adverse effect.

The reviewed classification systems in oncology are all tailored to specific contexts of use: JCR, NCT and ESCAT evaluate clinical utility with an emphasis on predictive genetic biomarkers (including biomarkers of resistance except ESCAT), but also, to a lesser extent, diagnostic and prognostic biomarkers. In addition, CIViC encompasses susceptibility/risk biomarkers as well. Finally, the EDRN framework was originally developed to provide a roadmap for useful population-cancer-screening susceptibility/risk and diagnostic biomarkers. None of these covers all seven categories.

Therefore, our unifying concept comprises: I. defining contexts of use, II. defining evidence levels and III. rating clinical utility (Fig. 1).

### Defining contexts of use

We introduce the context of use by grouping the biomarker into one of these seven categories and by defining the clinical endpoint which the biomarker is associated with (Fig. 1. I). This is a mandatory first step before any levels of evidence can be assigned to the biomarker to be classified. For example, Neurofilament light chain can be evaluated from different contexts of use: 1. As a prognostic biomarker in amyotrophic lateral sclerosis (clinical endpoint: change in ALS functional rating scale), 2. As a monitoring/pharmacodynamic response biomarker (for nusinersen response in spinal muscular atrophy type 1 and natalizumab response in MS), 3. As a diagnostic biomarker (clinical endpoint: differential diagnosis between Parkinson's disease and atypical Parkinsonian disorders), as well as several more (see Supplementary Tables S1–S4 and Supplementary Files S2–S5).

### Defining evidence levels

The classification of evidence levels within our unified concept should be applicable for all potential contexts of use and all types of molecular biomarkers. Proceeding from the reviewed classification systems in oncology, we decided to omit oncology—and genetic-specific categories (such as ESCAT I C: basket trials and trials across disease types, ESCAT III B: alteration with predicted impact in same pathway as I, JCR Tier 3: no convincing evidence of cancer association, NCT m2 A–C: different entity).

Moreover, we advise to think of achieving analytical validity and clinical validity as two separate processes, although, naturally, they are intertwined in biomarker development. The rating of analytical validity is dependent on the molecular subtype of the biomarker and the method by which it is measured. While this is beyond the scope of this manuscript, several reviews have been published.^18^, ^19^, ^20^ Concerning the EDRN roadmap, phase 5 disease burden reduction studies cannot be accomplished for all contexts of uses of biomarkers—for example, in view of orphan diseases with no disease-modifying treatment available so far, this approach would lead to lower levels of evidence that not always reflect clinical utility.

Finally, we decided not to take regulatory approval and clinical guidelines into account. Devising these usually constitutes a lengthy procedure so that parallel-accumulating evidence is often not incorporated.

Therefore, we base the classification of evidence levels within our unified concept on the easier to apply and more general CIViC evidence levels. We further stratify the broad CIViC level B (clinical trial or other primary patient data supports association) into three levels (Fig. 1. II, Panel 1):

• B1. Prospective, randomized clinical trial
• B2. Cross-sectional and longitudinal cohort biomarker studies with de novo data collection and prespecified study protocol comprising the biomarker of interest
• B3. Retrospective biomarker studies

In this way, we consider aspects of clinical study design of high relevance to rating the actual added value of a study for routine clinical use—as introduced by the NCT, ESMO and EDRN classification systems. We propose that level A evidence (proven/consensus association in human medicine) can only be achieved by validation of appropriate B1 or B2 studies in at least 1 independent cohort employing the same methods (or one meta-analysis comprising such studies). Robustness of the analytical measurement techniques and thresholds in concentration must be achieved. Factors determining variation of a biomarker in the healthy population must be delineated. Inclusion of the context of use of the biomarker to be classified in guidelines is not necessary but underscores the validity of level A biomarkers.

### Rating clinical utility

How to translate the evidence levels within a specified context of use as introduced by our unified concept into actual clinical utility heavily depends on the individual context of use and cannot be generalized (Fig. 1. III).

In general, evidentiary standards that need to be addressed for clinical biomarker qualification depend on tolerability of risk in conjunction with its very specific context of use (i.e. consequences of false-positives and false-negatives).^15^ For example, a diagnostic or susceptibility/risk biomarker used as a screening tool for millions of individuals may require multiple replicate studies (randomized clinical trials are ruled out, instead the gold standard is a longitudinal investigation of disease incidence in a disease-negative cohort). In contrast, high evidence levels might never be accomplished for low incidence biomarkers such as those associated with orphan diseases. We decided not to disclose a recommended number of participants due to such different contexts of use.

Treatment related, such as pharmacodynamic/response and predictive biomarkers, are ideally prospectively investigated as part of a randomized controlled trial with biomarker-positive and -negative patients. Randomized trials, however, are expensive and time consuming, do not capture long-term effects, cannot be completely masked, and require that treatment implications of biomarker results are well defined. In addition, randomization might not be considered ethical when there is evidence of strong superiority of a compound. This may be why most clinical studies evaluating predictive biomarkers were conducted retrospectively.^15^^,^^21^ Sufficient specificity is crucial regarding predictive biomarkers not to withhold any patients from therapies.

It is outside the scope of this review to provide a detailed discussion of how to rate the design of a clinical study. We refer to an excellent review^22^ published in 2020 that deals with various aspects such as cohort related factors, relevant comparisons with state-of-the art methods and statistical analyses. It is difficult to evaluate whether statistical methods used in biomarker studies such as decision-analysis modelling or Bayesian baskets are correctly implemented. We decided to keep our classification of evidence levels simple, while being aware that rating the quality of a (clinical) study requires high expertise. Regarding the requirements of a biomarker to become a surrogate endpoint by itself, there even is no consensus among statistics experts.^15^^,^^23^

## Application of the unifying classification to selected neurologic disease entities

In the following, we aim to apply our classification system to biomarker studies using proteomic tools for the detection of established as well as promising future protein biomarkers for neurovascular, neuroinflammatory, neurooncologic and neurodegenerative diseases with a focus on the most frequent entities, respectively. Studies using human blood and CSF were included except for neurooncologic diseases where tumour tissue is also considered. Comprehensive lists of selected reviewed protein biomarkers are provided in the Supplementary Files S1–S5.

### Protein biomarkers of neurooncologic diseases

**Fig. 2:**
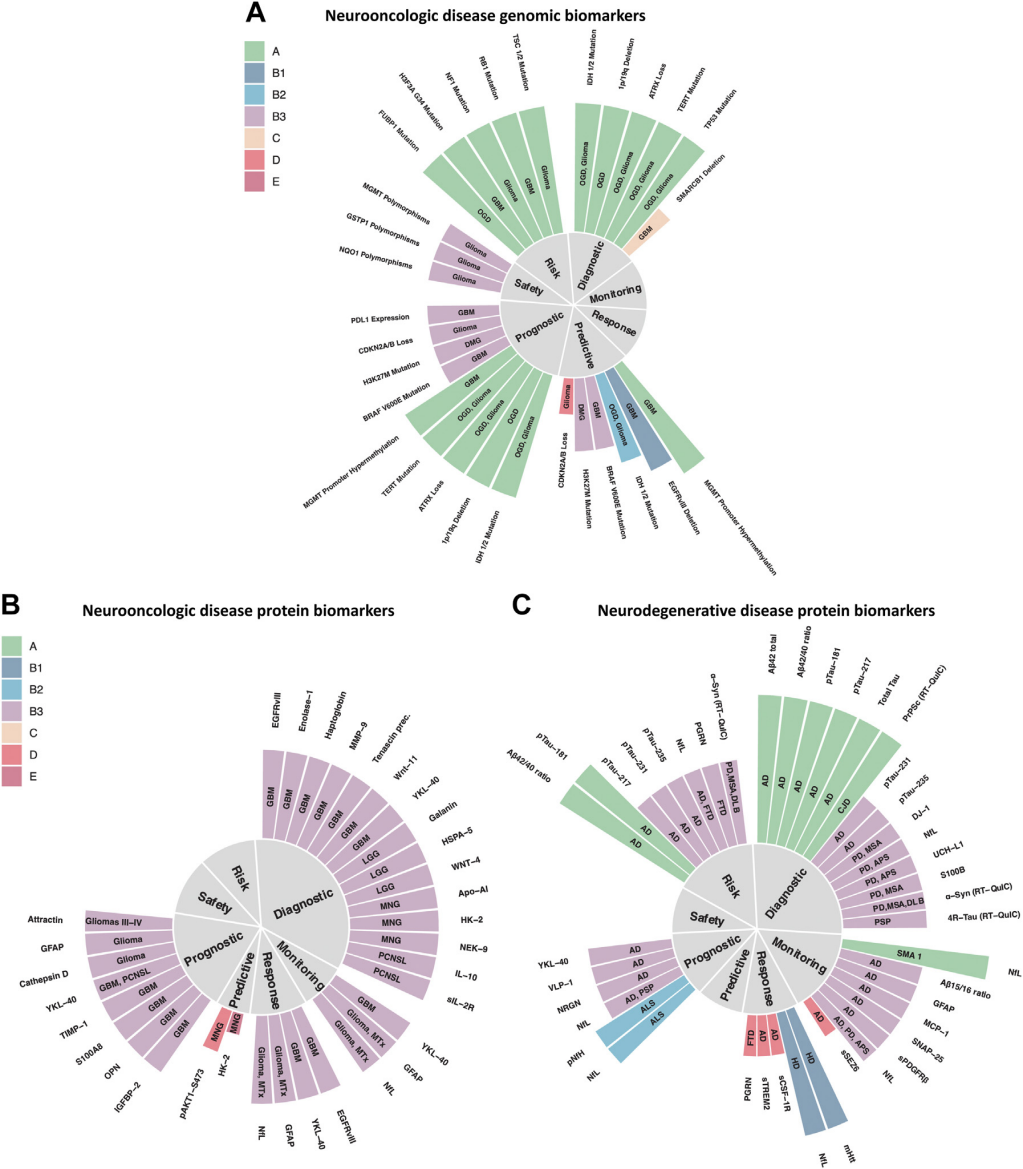

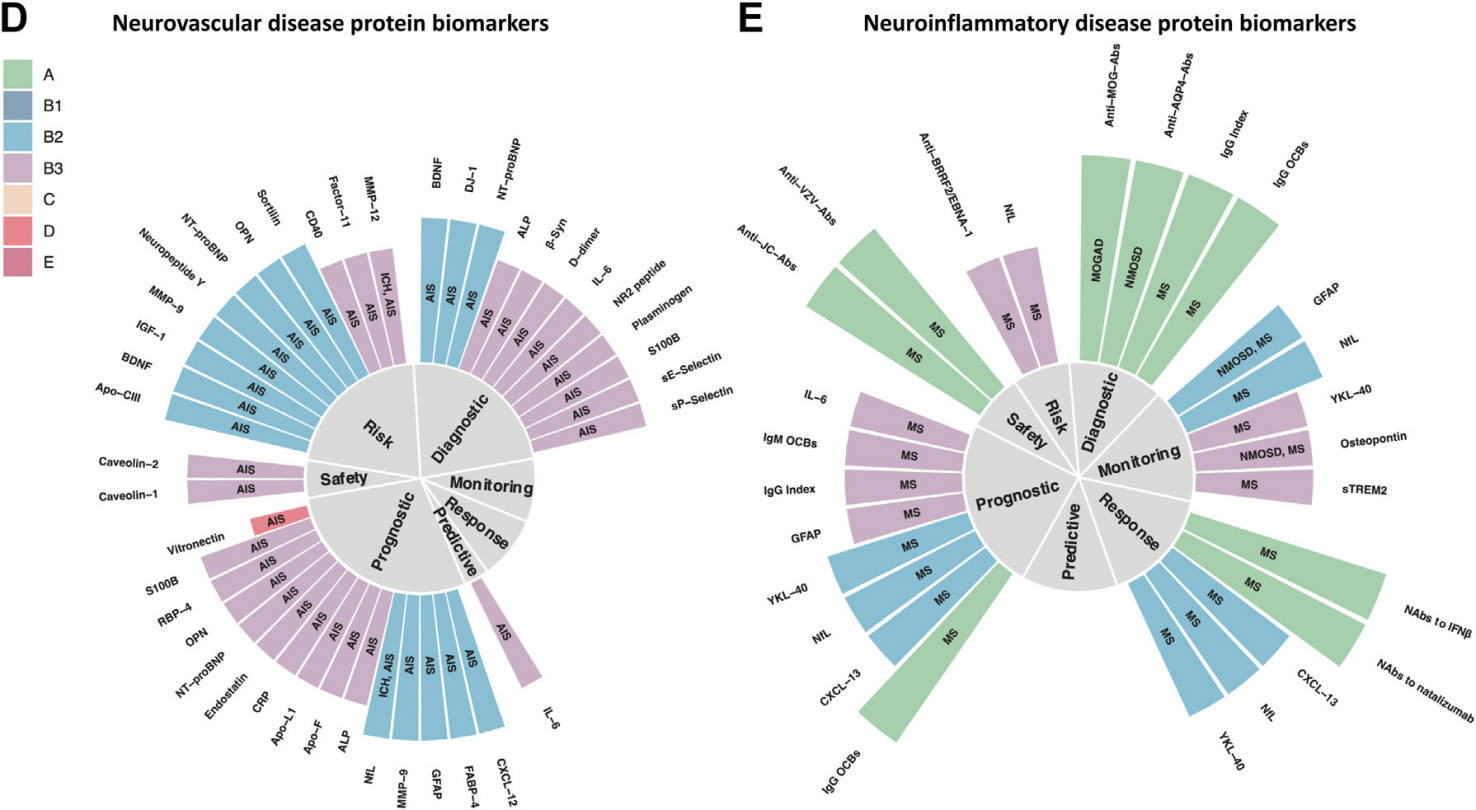
**Selected genomic biomarkers of neurooncologic (A) and selected protein biomarkers of neurooncologic (B), neurodegenerative (C), neurovascular (D) and neuroinflammatory (E) diseases.** Biomarkers were grouped into one of the seven categories of clinical applications and the corresponding evidence level is indicated by both bar length and color. Biomarkers with level A and B evidence form the basis, few biomarkers of lower evidence levels are shown as well. Disease entity is depicted by the signs within the bars. Abs (antibodies), AIS (Acute Ischemic Stroke), ALP (Alkaline Phosphatase), ALS (Amyotrophic Lateral Sclerosis), APS (Atypical Parkinsonian Syndromes), C1s (Complement component C1s), C5 (Complement component 5), CJD (Creutzfeldt-Jakob Disease), DLB (Dementia with Lewy Bodies), DMG (Diffuse Midline Glioma), FTD (Frontotemporal Dementia), GBM (Glioblastoma), HD (Huntington's Disease), ICH (Intracerebral Hemorrhage), LGG (Lower Grade Glioma), MNG (Meningeoma), MOGAD (Myelin Oligodendrocyte Glycoprotein antibody associated Autoimmune Disease), MS (Multiple Sclerosis), MSA (Multiple System Atrophy), MTx (brain metastases), AD (Alzheimer's Disease), Nabs (Neutralizing antibodies), NfL (Neurofilament light chain), NMOSD (Neuromyelitis Optica Spectrum Disorder), OCBs (Oligoclonal Bands), OGD (Oligodendroglioma), OPN (Osteopontin), PCNSL (Primary Central Nervous System Lymphoma), pNfH (phosphorylated Neurofilament heavy chain), PSP (Progressive Supranuclear Palsy), RT-QuIC (Real-Time Quaking-Induced Conversion).

Level A molecular biomarkers of neurooncologic diseases comprise solely genetic biomarkers: the 2016 and the 2021 WHO classification of CNS tumours^24^ pioneered molecular diagnostics–1p/19q codeletion and IDH mutation status were even introduced as tumour entity defining features, for example. Given the diagnostic and therapeutic role of surgery, these are assessed on tumour tissue. Regarding glioblastoma, some tissue biomarkers have shown to harbour level B3 prognostic (GFAP or estrogen receptor alpha^25^) or diagnostic value (for example, high expression of Wnt 11, Tenascin precursor or Enolase 1^26^ indicating high WHO grade). An elegant approach to circumvent an inherent limitation of blood-based biomarkers—that they often reflect systemic response processes to the tumour—was to measure extracellular vesicles in plasma carrying the neoantigen EGFRvIII^27^ as diagnostic and response (to temozolomide and geldanamycin) biomarkers. Level B3 prognostic biomarkers for survival measured in serum include YKL-40,^28^ IGFBP-2,^29^ TIMP1^30^ and osteopontin.^31^ There is level B3 evidence for serum YKL-40^32^ in glioblastoma as well as GFAP and NfL^33^ in various brain tumours for monitoring radiographic disease status. Radiographic features still form the basis for assessing whether a therapeutic response has occurred.^34^ More sensitive protein (or any molecular) response and monitoring biomarkers are lacking to differentiate true tumour progression from pseudo-tumour progression. Differentiating WHO grades of meningioma could be achieved by measuring apolipoproteins A–I, J, E and hemopexin in a level B3 study as diagnostic biomarkers.^35^ The same study identified pAKT1-S473 and HK2 as potential predictive biomarkers. Nevertheless, none of the mentioned protein biomarkers seem to be ready for clinical implementation (Fig. 2A and B, Supplementary Table S1, and Supplementary Files S1 and S2).

### Protein biomarkers of neurodegenerative diseases

Level A protein biomarkers comprise those integrated in the current diagnostic criteria for AD^2^ that provide an *in vivo* estimate of the presence of Alzheimer's pathology: CSF markers of amyloid pathology (Aβ42/40 ratio, Aβ42^36^), markers reflecting tau pathophysiology (phospho-tau species 181^37^ and 217^38^) and neuro(axo)nal damage (total-tau) according to the ATN criteria. The latter biomarker is likely to be replaced by neurofilament light, which is more strongly linked to neurodegeneration than total-tau.^39^ Protein biomarkers of amyloid- and tau-related pathologies can be used for risk stratification in asymptomatic individuals and their presence indicates a higher risk for a conversion of mild cognitive impairment to AD. The ATN criteria are starting to provide a biological definition of AD. This (r)evolution to clinico-biological diagnosing is underway in the second most common neurodegenerative disorder—Parkinson's disease (PD) as well as atypical parkinsonian syndromes where a purely clinical diagnosing still is the gold-standard.^40^, ^41^, ^42^, ^43^ Biomarkers of protein aggregate pathology such as 3R/4R-tau real-time-quaking-induced-conversion (RT-QuIC)^44^ and alpha-synuclein RT-QuIC^45^ are probably the most sensitive and specific single diagnostic biomarkers for the determination of underlying pathology. They are promising susceptibility/risk biomarkers in individuals with prodromal disease stages, such as rapid eye movement sleep behaviour disorder for synucleinopathies.^46^ In contrast, various prognostic and monitoring biomarkers often reflect more or less unspecific biological pathways. For AD, these include protein biomarkers of synaptic degeneration (such as neurogranin,^47^ SNAP-25^48^), microglial and astroglial activation (YKL-40,^49^ GFAP,^50^ sTREM2^51^) as well as neuro(axo)nal damage (NfL^52^^,^^53^). Nevertheless, these may prove useful for further stratifying molecular disease subtypes that are relevant to prognosis and possibly response to future disease-modifying therapies. Regarding PD, NfL,^54^ UCH-L1^55^ and DJ-1^56^ have shown some potential as biomarkers facilitating differential diagnosis between PD and atypical PD at group level, although their informative value for the respective individual remains unclear. Most of the cited studies for prognostic and monitoring protein biomarkers are retrospective cross-sectional biomarker studies (level B3). Prospective (level B2) and longitudinal data are available for only few protein biomarkers, such as NfL and pNfH in ALS^57^^,^^58^ or Aβ42/40 ratio, phospho-Tau181 and 217 in AD. Given the lack of disease-modifying therapies for neurodegenerative diseases, very few treatment-related biomarkers have been investigated in patients. NfL in SMA type 1 may serve as a pharmacodynamic/response biomarker (nusinersen, level A^59^). In more common neurodegenerative diseases, such as AD, protein treatment-related biomarkers display low levels of evidence as no disease-modifying therapies are available (apart from the recently FDA-approved aducanumab^60^). This especially applies to safety and predictive biomarkers. Establishing biomarker assays using more easily available specimen such as blood, tears, saliva, or urine instead of invasive lumbar punctures is an important future direction, but harbours significant challenges. Aβ blood tests,^61^^,^^62^ for example, lack robustness due to extra-cerebral sources of Aβ and a more pronounced diurnal variation than what has been seen for CSF.^63^ Aβ, alpha-synuclein, total tau, and phospho-tau species can be detected in saliva. Lactoferrin has potential for detection of early AD.^64^ However, standardization of collection (stimulated vs unstimulated) and storage methods as well as clear ranges for diagnosis are needed (Fig. 2C, Supplementary Table S2, and Supplementary File S3).

### Protein biomarkers of neurovascular diseases

The majority of protein biomarkers for neurovascular diseases show level B2-3 evidence with no biomarker showing level A/B1, overall mirroring that there is no protein biomarker with accepted clinical value for stroke and associated neurovascular diseases.^65^ While circulating levels of several proteins are assessed in the acute phase of stroke to detect systemic complications of stroke (e.g. CRP, GOT, GPT^66^), there is no circulating protein that supports clinical decisions by reliably detecting local pathophysiological processes in stroke, e.g. vessel occlusion, (ischemic) neuro(axo)nal injury, blood–brain barrier dysfunction, and the local immune response. Possible reasons are i) investigated proteins not meeting the requirements to support clinical decisions (e.g. delayed detection of neuronal injury by S100B^67^ and NfL^68^ limiting their value in supporting time-sensitive treatment decisions in the acute phase of stroke) and ii) lack of studies supporting high-level evidence of protein biomarkers (e.g. a randomized controlled trial for an NT-proBNP-based decision on secondary stroke prevention). Protein biomarkers of stroke risk might guide decisions on primary stroke prevention and have been shown to inform on stroke risk beyond clinical scales in secondary analyses of RCT data (e.g. NT-proBNP beyond the CHA2DS2VASc score^69^). However, whether a primarily biomarker-based or a biomarker-supported clinical scale is superior to established clinical scales alone to prevent stroke has not been assessed in a randomized controlled trial. The third group of protein biomarkers with a high number of studied proteins in neurovascular diseases are prognostic biomarkers (Fig. 2D). Many proteins have been demonstrated to show value to predict long-term outcome (mostly functional outcome assessed by the modified Rankin Scale score at 3 months after stroke) beyond initial symptom severity, e.g. MMP9,^70^ GFAP,^71^ NfL,^68^ S100B.^67^ However, in the vast majority of studies it remains unclear which specific clinical decision can be supported by the identified prognostic value—a question that should ideally be taken into account when designing the study (Fig. 2D, Supplementary Table S3, and Supplementary File S4).

### Protein biomarkers of neuroinflammatory diseases

The revised McDonald criteria^3^ provide a blueprint for neuroimaging-based clinico-biological diagnosing: when clinical evidence is lacking, magnetic resonance imaging (MRI) findings can serve as surrogates for dissemination in space and/or time to diagnose MS. The hallmark of typical CSF changes in MS is increased production of intrathecal immunoglobulins as shown by quantitatively elevated IgG and/or detection of oligoclonal bands (OCB). The revised McDonald criteria underline the importance of CSF OCBs as a level A diagnostic and prognostic biomarker that can substitute for demonstration of dissemination of lesions in time in some settings. Although they are elevated in a number of chronic inflammatory diseases, their absence harbours a negative predictive value of about 90%^72^—even in clinically isolated syndrome (CIS).^73^ To a lesser extent, the above also applies to IgG index.^3^^,^^74^^,^^75^ Intrathecal IgG and IgM synthesis show some prognostic value in MS as their presence has been associated with disease progression (Fig. 2E, Supplementary Table S4, and Supplementary file S5).^76^^,^^77^

OCBs appear significantly less frequent in Neuromyelitis optica spectrum disorders (NMOSD) and MOG-IgG antibody associated autoimmune disease (MOGAD)—to about 13%.^78^ In the case of NMOSD, antibodies against aquaporin 4 (AQP4)^79^^,^^80^ are helpful level A diagnostic biomarkers, whereas high antibody titer against MOG are defining level A diagnostic biomarkers for MOGAD.^78^^,^^81^ Compared to MS, NMOSD displays elevated amounts of GFAP in CSF and serum.^82^

Promising disease monitoring, response and prognostic biomarkers include those that reflect more or less unspecific damage and repair processes, such as: GFAP as a surrogate marker for astrocyte damage,^83^ sTREM2 as a selective marker of microglial activation,^84^ the not (only) brain-derived chemokine CXCL13—a selective chemoattractant for B lymphocytes and follicular B helper T cells^85^ as well as the cytokines IL6 and IL10, and NfL as a marker for axonal damage.^86^^,^^87^ Serum NfL displays potential as monitoring and response biomarker for treatment with disease modifying therapies in MS and NMOSD.^87^^,^^88^ Neutralizing antibodies (such as against natalizumab^89^ and interferon beta^90^) are level A response MS biomarkers indicating loss of efficacy. Level A safety biomarkers include for example antibodies against John Cunningham Virus (JCV) that predict the development of progressive multifocal leukoencephalopathy (PML) in MS patients. Biomarkers to stratify MS subtypes and predict risk of developing MS in asymptomatic patients are urgently needed.

Biomarkers may also help to identify persons at risk for MS. The presence of antibodies against EBV, in particular EBNA1, is associated with increased MS risk.^91^ Also genetic variants and HLA alleles have been identified, which allow to determine an individual polygenic risk score.^92^ The combination of these and other biomarkers may help to identify patients at risk to facilitate early diagnosis and treatment.

## Conclusion and outlook

The informative value of a protein biomarker for individual patients is still unclear, as most studies have addressed their behaviour on the level of entire populations. sNfL percentiles and z-scores have recently been published in the context of MS^87^ and outperformed absolute raw cut-off values. Large longitudinal level B2 studies in both patients and healthy individuals are needed to retrieve individual reference values controlling for potential covariates such as age, sex, and BMI. Longitudinal studies were also crucial to determine half-life of NfL blood concentrations, which are different compared to tau (10 days vs several months), and has impact for its value as a monitoring biomarker for diseases occurring at different time scales.^93^ In general, within-individual changes of potential clinical significance may be concealed by between-individuals variation of biomarker test results as mostly cross-sectional retrospective studies have been performed.

An important caveat concerning the interpretation of many (protein) biomarkers is the lack of biomarker studies with clinicopathologic correlation, which leads to uncertainness of clinical diagnoses. This applies, above all, to diseases where brain biopsies are usually not an integral part of the diagnostic process, such as neurodegenerative, neurovascular and neuroinflammatory diseases. For instance, many neurodegenerative proteinopathies can be clinically indistinguishable and one single proteinopathy can cause multiple different clinical phenotypes.^94^ For example, the clinical phenotype “corticobasal syndrome” can be caused by PSP, CBD or AD pathologies, less frequently by depositions of TDP-43. Furthermore, co-pathologies such as TDP-43- or α-synucleinopathy and coexistent cerebrovascular pathologies are frequently present.^95^, ^96^, ^97^ Nonetheless, Biomarker studies involving autopsy confirmation meet inherent challenges: biomarker measurements reflect the very latest stages of the disease, and may be affected by the lag time between death and autopsy, especially for proteins that are highly labile, and sample sizes are often relatively small.

An evolving issue in view of large-scale molecular stratification efforts by high-throughput and multiplexed methods are composite biomarkers—extending the concept from single affected biomarkers to combinations of these. For example, tumour mutational burden, defined as the number of somatic mutations per megabase of interrogated genomic sequence, is recognised as a predictive biomarker for immunotherapy outcomes. In AD, an 18 plasma protein panel has been discovered to differentiate blinded samples from AD and control subjects with close to 90% accuracy.^98^ However, a replication study^99^ only yielded an accuracy of 63%. Similarly, only nine out of 94 previously described blood biomarkers could be validated by a replication study.^100^ Lack of reproducibility of data is a major concern^101^ as most of the effort has been directed towards biomarker discovery studies, often related to small sample sizes and the retrospective study design. In this regard, publication bias may be an issue as negative or failed biomarker (replication) studies may be underpublished. An up-to-date open access platform for sharing biomarker data of patient cohorts and healthy individuals as well as real-world data would be desirable.

Characterizing molecular patterns of tumours led to the approval of several targeted cancer therapies in molecular biomarker-stratified clinical trials. There is a long way to go until precision medicine becomes feasible for many of the frequent neurologic diseases that contribute so much to loss in disability-adjusted life-years, such as Stroke, MS or neurodegenerative proteinopathies like AD. We highlighted lack of evidence for many protein biomarkers in neurology with few prospective or even randomized clinical data available. We proposed a unified classification system that may help to identify promising biomarker candidates and provide a roadmap to increase levels of evidence of existing biomarkers.

**Search strategy and selection criteria**

We considered national and international guidelines, reviews, and other articles, listed at NCBI-PubMed, which contain listings of (potential) protein biomarkers. Proceeding from these listings, (potential) biomarkers were further reviewed by searching PubMed for the following terms: “potential biomarker” AND “associated disease”. Due to the high number of alias names of the term “potential biomarker” and “associated disease”, the specific MeSH-terms used by the PubMed search engine are not listed here explicitly. Studies using human tissue, blood and CSF were included. For a more detailed discussion of biomarker candidates, selected in vitro and animal-based studies were considered as well. We also searched the references within the selected papers for relevant articles. We reviewed only papers in English. We did not apply date restrictions to the search. The last search was done on Mar 31, 2022. The final reference list was generated on the basis of relevance to the topics covered in this Review.

## Contributors

Alexander M. Bernhardt: Conceptualization, Methodology, Investigation, Data curation, Writing - Original Draft and coordination of neurodegenerative diseases section, Writing - Review & Editing, Visualization, Project administration.

Steffen Tiedt: Investigation, Writing - Review & Editing, Writing - Original Draft - coordination of neurovascular diseases section.

Daniel Teupser: Writing - Review & Editing, Funding acquisition, Supervision.

Martin Dichgans: Writing - Review & Editing, Funding acquisition, Supervision, Writing - Original Draft - coordination of neurovascular diseases section.

Bernhard Meyer: Writing - Review & Editing, Funding acquisition, Supervision, Writing - Original Draft - coordination of neurooncologic diseases section.

Jens Gempt: Investigation, Writing - Review & Editing, Writing - Original Draft - coordination of neurooncologic diseases section.

Peer-Hendrik Kuhn: Investigation, Writing - Review & Editing, Writing - Original Draft - coordination of neurooncologic diseases section.

Mikael Simons: Writing - Review & Editing, Funding acquisition, Supervision, Writing - Original Draft - coordination of neuroinflammatory diseases section.

Carla Palleis: Investigation, Writing - Review & Editing.

Endy Weidinger: Investigation, Writing - Review & Editing.

Georg Nübling: Investigation, Writing - Review & Editing.

Lesca Holdt: Investigation, Writing - Review & Editing.

Lisa Hönikl: Investigation, Writing - Review & Editing.

Christiane Gasperi: Investigation, Writing - Review & Editing.

Pieter Giesbertz: Investigation, Writing - Review & Editing.

Stephan A. Müller: Investigation, Writing - Review & Editing.

Stephan Breimann: Investigation, Writing - Review & Editing.

Stefan F. Lichtenthaler: Writing - Review & Editing, Funding acquisition, Supervision.

Bernhard Kuster: Writing - Review & Editing, Funding acquisition, Supervision.

Matthias Mann: Writing - Review & Editing, Funding acquisition, Supervision.

Axel Imhof: Writing - Review & Editing, Funding acquisition, Supervision.

Teresa Barth: Investigation, Writing - Review & Editing.

Stefanie M. Hauck: Writing - Review & Editing, Funding acquisition, Supervision.

Henrik Zetterberg: Writing - Review & Editing, Supervision.

Markus Otto: Writing - Review & Editing, Supervision.

Wilko Weichert: Conceptualization, Methodology, Writing - Review & Editing, Funding acquisition, Supervision, Writing - Original Draft - coordination of neurooncologic diseases section.

Bernhard Hemmer: Conceptualization, Methodology, Writing - Review & Editing, Writing - Original Draft - coordination of neuroinflammatory diseases section, Funding acquisition, Supervision.

Johannes Levin: Conceptualization (lead), Methodology (lead), Supervision (lead), Writing - Review & Editing, Writing - Original Draft and coordination of neurodegenerative diseases section, Funding acquisition (lead).

All authors read and approved the final version of the manuscript, and decided to submit the manuscript to EBioMedicine together.

## Declaration of interests

Johannes Levin reports part-time employment by MODAG GmbH and a grant of the Michael J Fox Foundation for Parkinson's Research. In addition, he reports speaker fees from Bayer Vital, Biogen and Roche, consulting fees from Axon Neuroscience and Biogen, author fees from Thieme medical publishers and W. Kohlhammer GmbH medical publishers, all outside the submitted work. He is a member of the advisory board of Biogen and a member of the Data Safety Monitoring Board of Axon Neuroscience. He is beneficiary of the phantom share program of MODAG GmbH. In addition, he is inventor in a patent “Pharmaceutical Composition and Methods of Use” (EP 22 159 408.8) filed by MODAG GmbH.

Bernhard Hemmer received funding by the European Union's Horizon 2020 Research and Innovation Program and the Deutsche Forschungsgemeinschaft (DFG, German Research Foundation) under Germany's Excellence Strategy within the framework of the Munich Cluster for Systems Neurology and Roche. He holds part of two patents: one for the detection of antibodies against KIR4.1 in a subpopulation of patients with multiple sclerosis and one for genetic determinants of neutralizing antibodies to interferon.

Wilko Weichert reports research funding from Roche, MSD, BMS and AstraZeneca. He has attended and given talks at Advisory Boards, gave advice to and served as speaker on national and international conferences for Roche, MSD, BMS, AstraZeneca, Pfizer, Merck, Lilly, Boehringer, Novartis, Takeda, Bayer, Amgen, Astellas, Eisai, Johnson and Johnson, Janssen, Illumina, Siemens, Agilent, ADC, GSK and Molecular Health.

Stefan F. Lichtenthaler reports research funding from Shionogi and Novartis.

Steffen Tiedt reports consulting fees from Alpha Apollo Inc.

Christiane Gasperi reports funding from the Hertie Foundation, the Deutsche Forschungsgemeinschaft (DFG, German Research Foundation) and the Hans and Klementia Langmatz Stiftung.

Carla Palleis reports funding from the Deutsche Forschungsgemeinschaft (DFG, German Research Foundation) under Germany's Excellence Strategy within the framework of the Munich Cluster for Systems Neurology.

No other disclosures were reported.
