# Supplementary material for: A unified classification approach rating clinical utility of protein biomarkers across neurologic diseases
Source: eBioMedicine. 2023 Feb 4;89:104456. doi: 10.1016/j.ebiom.2023.104456 (PMC9931915; doi:10.1016/j.ebiom.2023.104456)
Supplement: Supplementary Tables S1–S4 [file mmc1.docx]

**Supplementary table 1: Unified classification system applied to selected protein neuro-oncologic disease biomarkers**

| **Name of biomarker** | **UniProt-KB or LOINC number** | **Type of specimen** | | **Associated disease** | **Clinical application of biomarker** | | | **Associated clinical endpoint** | **Level of evidence** | | **References** | |  |
| --- | --- | --- | --- | --- | --- | --- | --- | --- | --- | --- | --- | --- | --- |
| **Cathepsin D** | P07339 (CATD_HUMAN) | Serum | Glioma | | | Prognostic | Overall survival | | | B3 | | [1] | |
| **EGFRvIII** | Not available | Plasma (microvesicles) | Glioblastoma | | | Diagnostic | Glioblastoma  (clinical diagnosis) | | | B3 | | [2] | |
| **Glial fibrillary acidic protein** | P14136 (GFAP_HUMAN) | CSF | Glioma | | | Prognostic | Overall survival,  tumour size | | | B3 | | [3] | |
| **Haptoglobin** | P00738 (HPT_HUMAN) | Serum | Glioblastoma | | | Diagnostic | Differential diagnosis: indicator for glioblastoma | | | B3 | | [4] | |
| **Matrix metalloproteinase-9** | P14780 (MMP9_HUMAN) | CSF, Serum | Glioblastoma | | | Diagnostic | Differential diagnosis: indicator for glioblastoma | | | B3 | | [5] | |
| **Osteopontin** | P10451 (OSTP_HUMAN) | Serum | Glioblastoma | | | Prognostic | Overall survival | | | B3 | | [6] | |
| **pAKT1-S473** | Not available | Meningeoma cell line | Meningeoma | | | Predictive | Response to pan-AKT kinase inhibitor AZD5363 | | | D | | [7] | |
| **YKL-40/chitinase-3-like protein 1 (CHI3L1)** | P36222 (CH3L1_HUMAN) | CSF, Serum | Glioblastoma | | | Monitoring | Radiographic disease status (MRI) | | | B3 | | [8] | |
| **YKL-40/chitinase-3-like protein 1 (CHI3L1)** | P36222 (CH3L1_HUMAN) | CSF | Glioma, Brain metastases, Lymphoma | | | Prognostic | Overall survival,  tumour size | | | B3 | | [3,9] | |
| **α-2-Heremans-Schmid glycoprotein (AHSG)** | P02765 (FETUA_HUMAN) | Serum | Glioblastoma | | | Prognostic | Overall survival | | | B3 | | [10] | |

**Supplementary table 2: Unified classification system applied to selected protein neurodegenerative disease biomarkers**

| **Name of biomarker** | | **UniProt-KB or LOINC number** | | **Type of specimen** | | **Associated disease** | | **Clinical application** | **Associated clinical endpoint** | **Level of evidence** | **References** |
| --- | --- | --- | --- | --- | --- | --- | --- | --- | --- | --- | --- |
| **Aβ42/40 ratio** | | LOINC: 98485-6 | | CSF | | Alzheimer's disease | Diagnostic | Alzheimer's disease (clinical diagnosis) | A | [11,12] |  |
| **Aβ42/40 ratio** | | Not available | | Plasma | | Alzheimer's disease | Diagnostic | Alzheimer's disease (clinical diagnosis) | B2 | [13,14] |  |
| **Glial fibrillary acidic protein** | | P14136 (GFAP_HUMAN) | | Serum | | Alzheimer's disease | Monitoring | Cognitive decline | B3 | [15] |  |
| **Neurofilament light chain** | | P07196 (NFL_HUMAN) | | CSF | | Spinal muscular atrophy type 1 | Monitoring | Response to nusinersen treatment | A | [16] |  |
| **Neurofilament light chain** | | P07196 (NFL_HUMAN) | | Serum | | Multiple system atrophy | Monitoring | Disease severity | B3 | [17] |  |
| **Neurofilament light chain** | | P07196 (NFL_HUMAN) | | CSF, Serum, Plasma | | Parkinson's disease, Multiple system atrophy, Progressive supranuclear palsy | Diagnostic | Differential diagnosis Parkinson's disease vs. atypical Parkinsonian syndromes | B3 | [18,19] |  |
| **Neurogranin** | | Q92686 (NEUG_HUMAN) | | CSF | | Alzheimer's disease | Prognostic | Cognitive decline | B3 | [20] |  |
| **Progranulin** | | P28799 (GRN_HUMAN) | | Plasma | | Genetic frontotemporal dementia (GRN+) | Susceptibility/risk | Risk for developing clinical frontotemporal dementia | B3 | [21] |  |
| **sSEZ6 (solulable seizure 6 protein)** | | Q53EL9 (SEZ6_HUMAN) | | Murine CSF | | Alzheimer's disease | Monitoring | Monitoring application of BACE1-Inhibitors | D | [22] |  |
| **sTREM2 (triggering receptor expressed on myeloid cells 2)** | | Q9NZC2  (TREM2_HUMAN) | | Murine CSF | | Alzheimer's disease | Pharmacodynamic/ response, Monitoring | AL002 treatment | D | [23] |  |

**Supplementary table 3: Unified classification system applied to selected protein neurovascular disease biomarkers**

| **Name of biomarker** | **UniProt-KB or LOINC number** | **Type of specimen** | **Associated disease** | **Clinical application** | **Associated clinical endpoint** | **Level of evidence** | **References** |
| --- | --- | --- | --- | --- | --- | --- | --- |
| **Glial fibrillary acidic protein** | P14136 (GFAP_HUMAN) | Serum | Ischemic stroke | Prognostic | Outcome (NIHSS) | B2 | [24] |
| **Matrix metalloproteinase 12** | P39900 (MMP12_HUMAN) | Serum | Ischemic stroke | Susceptibility risk | Ischemic stroke (etiology subtype left atrial appendage) | B3 | [25] |
| **Matrix metalloproteinase 9** | P14780 (MMP9_HUMAN) | Serum | Ischemic stroke | Prognostic | Mortality, major disability (Modified Rankin Scale) | B2 | [26] |
| **Neurofilament light chain** | P07196 (NFL_HUMAN) | Serum | Ischemic stroke, intracerebral hemorrhage | Prognostic | Mortality, outcome (NIHSS, Modified Rankin Scale) | B3 | [27,28] |
| **Neuropeptide Y** | P01303 (NPY_HUMAN) | Serum | Ischemic stroke | Susceptibility risk | Post-ischemic stroke epilepsy | B2 | [29] |
| **NT-proBNP** | P16860 (ANFB_HUMAN) | Serum | Ischemic stroke | Prognostic | Stroke onset after transitory ischemic attack | B3 | [30] |
| **Plasminogen** | P00747 (PLMN_HUMAN) | Serum | Ischemic stroke | Diagnostic | Stroke onset | B3 | [31] |
| **Retinol-Binding Protein 4** | P02753 (RET4_HUMAN) | Serum | Ischemic stroke | Prognostic | Outcome (NIHSS) | B3 | [32] |
| **S100B** | P04271 (S100B_HUMAN) | CSF | Ischemic stroke | Susceptibility risk | Post-ischemic stroke epilepsy | B3 | [33] |
| **sP-selectin** | P16109 (LYAM3_HUMAN) | Serum | Ischemic stroke | Diagnostic | Stroke onset | B3 | [34] |

**Supplementary table 4: Unified classification system applied to selected protein neuroinflammatory disease biomarkers**

| **Name of biomarker** | **UniProt-KB or LOINC number** | **Type of specimen** | **Associated disease** | **Clinical application** | **Associated clinical endpoint** | **Level of evidence** | **References** |
| --- | --- | --- | --- | --- | --- | --- | --- |
| **Neurofilament light chain** | P07196 (NFL_HUMAN) | Serum, Plasma, CSF | Multiple sclerosis | Pharmacodynamic response | Response to treatment with natalizumab  (Expanded Disability Status Scale) | B2 | [35] |
| **YKL-40/chitinase-3-like protein 1 (CHI3L1)** | P36222 (CH3L1_HUMAN) | CSF | Multiple sclerosis | Prognostic | Conversion from clinically isolated syndrome to multiple sclerosis | B2 | [36,37] |
| **YKL-40/chitinase-3-like protein 1 (CHI3L1)** | P36222 (CH3L1_HUMAN) | CSF | Multiple sclerosis | Monitoring | Disease activity | B2 | [36] |
| **CXC Chemokine Ligand 13** | O43927 (CXL13_HUMAN) | Serum, Plasma | Multiple sclerosis | Prognostic | Conversion to multiple sclerosis in patients with clinically isolated syndrome | B2 | [38] |
| **Glial fibrillary acidic protein** | P14136 (GFAP_HUMAN) | CSF, Serum | Multiple sclerosis | Monitoring | Expanded Disability Status Scale | B2 | [39,40] |
| **IgG Index** | LOINC: 48666-2, 68974-5, 42207-1 | Serum, Plasma, CSF | Multiple sclerosis | Prognostic | Associated with higher risk of conversion in multiple sclerosis when detected in clinically isolated syndrome (occurrence of new MRI lesions) | B3 | [41,42] |
| **Interleukin 6** | P05231 (IL6_HUMAN) | Serum, Plasma, CSF | Multiple sclerosis | Prognostic | Relapse frequency in female multiple sclerosis patients and age at onset for all multiple sclerosis patients | B3 | [43] |
| **Matrix metalloproteinase 9** | P14780 (MMP9_HUMAN) | CSF, Serum | Multiple sclerosis | Pharmacodynamic response | Response to treatment with natalizumab, interferon β | B2 | [44–46] |
| **Neutralizing antibodies to natalizumab** | LOINC: 49598-6, 58012-6 | Serum, Plasma | Multiple sclerosis | Pharmacodynamic response | Loss of natalizumab treatment efficacy | A | [47] |
| **sTREM2 (triggering receptor expressed on myeloid cells 2)** | Q9NZC2  (TREM2_HUMAN) | CSF | Multiple sclerosis | Monitoring | Expanded Disability Status Scale | B3 | [48] |

**References for supplementary tables 1-4**

[1] Fukuda ME, Iwadate Y, Machida T, Hiwasa T, Nimura Y, Nagai Y, et al. Cathepsin D is a potential serum marker for poor prognosis in glioma patients. Cancer Res 2005;65:5190–4. https://doi.org/10.1158/0008-5472.CAN-04-4134.

[2] Shao H, Chung J, Balaj L, Charest A, Bigner DD, Carter BS, et al. Protein typing of circulating microvesicles allows real-time monitoring of glioblastoma therapy. Nat Med 2012;18:1835–40. https://doi.org/10.1038/nm.2994.

[3] Schmid D, Warnken U, Latzer P, Hoffmann DC, Roth J, Kutschmann S, et al. Diagnostic biomarkers from proteomic characterization of cerebrospinal fluid in patients with brain malignancies. Journal of Neurochemistry 2021;158:522–38. https://doi.org/10.1111/jnc.15350.

[4] Kumar DM, Thota B, Shinde SV, Prasanna KV, Hegde AS, Arivazhagan A, et al. Proteomic Identification of Haptoglobin α2 as a Glioblastoma Serum Biomarker: Implications in Cancer Cell Migration and Tumor Growth. J Proteome Res 2010;9:5557–67. https://doi.org/10.1021/pr1001737.

[5] Hormigo A, Gu B, Karimi S, Riedel E, Panageas KS, Edgar MA, et al. YKL-40 and Matrix Metalloproteinase-9 as Potential Serum Biomarkers for Patients with High-Grade Gliomas. Clin Cancer Res 2006;12:5698–704. https://doi.org/10.1158/1078-0432.CCR-06-0181.

[6] Sreekanthreddy P, Srinivasan H, Kumar DM, Nijaguna MB, Sridevi S, Vrinda M, et al. Identification of Potential Serum Biomarkers of Glioblastoma: Serum Osteopontin Levels Correlate with Poor Prognosis. Cancer Epidemiol Biomarkers Prev 2010;19:1409–22. https://doi.org/10.1158/1055-9965.EPI-09-1077.

[7] Dunn J, Ferluga S, Sharma V, Futschik M, Hilton DA, Adams CL, et al. Proteomic analysis discovers the differential expression of novel proteins and phosphoproteins in meningioma including NEK9, HK2 and SET and deregulation of RNA metabolism. EBioMedicine 2019;40:77–91. https://doi.org/10.1016/j.ebiom.2018.12.048.

[8] Iwamoto FM, Hottinger AF, Karimi S, Riedel E, Dantis J, Jahdi M, et al. Serum YKL-40 is a marker of prognosis and disease status in high-grade gliomas. Neuro Oncol 2011;13:1244–51. https://doi.org/10.1093/neuonc/nor117.

[9] Qin G, Li X, Chen Z, Liao G, Su Y, Chen Y, et al. Prognostic Value of YKL-40 in Patients with Glioblastoma: a Systematic Review and Meta-analysis. Mol Neurobiol 2017;54:3264–70. https://doi.org/10.1007/s12035-016-9878-2.

[10] Petrik V, Saadoun S, Loosemore A, Hobbs J, Opstad KS, Sheldon J, et al. Serum alpha 2-HS glycoprotein predicts survival in patients with glioblastoma. Clin Chem 2008;54:713–22. https://doi.org/10.1373/clinchem.2007.096792.

[11] Dubois B, Villain N, Frisoni GB, Rabinovici GD, Sabbagh M, Cappa S, et al. Clinical diagnosis of Alzheimer’s disease: recommendations of the International Working Group. The Lancet Neurology 2021;20:484–96. https://doi.org/10.1016/S1474-4422(21)00066-1.

[12] Olsson B, Lautner R, Andreasson U, Öhrfelt A, Portelius E, Bjerke M, et al. CSF and blood biomarkers for the diagnosis of Alzheimer’s disease: a systematic review and meta-analysis. The Lancet Neurology 2016;15:673–84. https://doi.org/10.1016/S1474-4422(16)00070-3.

[13] Schindler SE, Bollinger JG, Ovod V, Mawuenyega KG, Li Y, Gordon BA, et al. High-precision plasma β-amyloid 42/40 predicts current and future brain amyloidosis. Neurology 2019;93:e1647–59. https://doi.org/10.1212/WNL.0000000000008081.

[14] Nakamura A, Kaneko N, Villemagne VL, Kato T, Doecke J, Doré V, et al. High performance plasma amyloid-β biomarkers for Alzheimer’s disease. Nature 2018;554:249–54. https://doi.org/10.1038/nature25456.

[15] Oeckl P, Halbgebauer S, Anderl-Straub S, Steinacker P, Huss AM, Neugebauer H, et al. Glial Fibrillary Acidic Protein in Serum is Increased in Alzheimer’s Disease and Correlates with Cognitive Impairment. J Alzheimers Dis 2019;67:481–8. https://doi.org/10.3233/JAD-180325.

[16] Olsson B, Alberg L, Cullen NC, Michael E, Wahlgren L, Kroksmark A-K, et al. NFL is a marker of treatment response in children with SMA treated with nusinersen. J Neurol 2019;266:2129–36. https://doi.org/10.1007/s00415-019-09389-8.

[17] Zhang L, Cao B, Hou Y, Gu X, Wei Q, Ou R, et al. Neurofilament Light Chain Predicts Disease Severity and Progression in Multiple System Atrophy. Movement Disorders 2022;37:421–6. https://doi.org/10.1002/mds.28847.

[18] Abdo WF, Bloem BR, Van Geel WJ, Esselink RAJ, Verbeek MM. CSF neurofilament light chain and tau differentiate multiple system atrophy from Parkinson’s disease. Neurobiology of Aging 2007;28:742–7. https://doi.org/10.1016/j.neurobiolaging.2006.03.010.

[19] Hansson O. A biomarker for differential diagnosis of parkinsonian disorder. Neurology 2017:9.

[20] Portelius E, Zetterberg H, Skillbäck T, Törnqvist U, Andreasson U, Trojanowski JQ, et al. Cerebrospinal fluid neurogranin: relation to cognition and neurodegeneration in Alzheimer’s disease. Brain 2015;138:3373–85. https://doi.org/10.1093/brain/awv267.

[21] Meeter LHH, Patzke H, Loewen G, Dopper EGP, Pijnenburg YAL, Minkelen R van, et al. Progranulin Levels in Plasma and Cerebrospinal Fluid in Granulin Mutation Carriers. DEE 2016;6:330–40. https://doi.org/10.1159/000447738.

[22] Pigoni M, Wanngren J, Kuhn P-H, Munro KM, Gunnersen JM, Takeshima H, et al. Seizure protein 6 and its homolog seizure 6-like protein are physiological substrates of BACE1 in neurons. Molecular Neurodegeneration 2016;11:67. https://doi.org/10.1186/s13024-016-0134-z.

[23] Wang S, Mustafa M, Yuede CM, Salazar SV, Kong P, Long H, et al. Anti-human TREM2 induces microglia proliferation and reduces pathology in an Alzheimer’s disease model. J Exp Med 2020;217:e20200785. https://doi.org/10.1084/jem.20200785.

[24] Liu G, Geng J. Glial fibrillary acidic protein as a prognostic marker of acute ischemic stroke. Hum Exp Toxicol 2018;37:1048–53. https://doi.org/10.1177/0960327117751236.

[25] Chong M, Sjaarda J, Pigeyre M, Mohammadi-Shemirani P, Lali R, Shoamanesh A, et al. Novel Drug Targets for Ischemic Stroke Identified Through Mendelian Randomization Analysis of the Blood Proteome. Circulation 2019;140:819–30. https://doi.org/10.1161/CIRCULATIONAHA.119.040180.

[26] Zhong C, Yang J, Xu T, Xu T, Peng Y, Wang A, et al. Serum matrix metalloproteinase-9 levels and prognosis of acute ischemic stroke. Neurology 2017;89:805–12. https://doi.org/10.1212/WNL.0000000000004257.

[27] Gendron TF, Badi MK, Heckman MG, Jansen-West KR, Vilanilam GK, Johnson PW, et al. Plasma neurofilament light predicts mortality in patients with stroke. Science Translational Medicine 2020;12:eaay1913. https://doi.org/10.1126/scitranslmed.aay1913.

[28] Uphaus T, Bittner S, Gröschel S, Steffen F, Muthuraman M, Wasser K, et al. NfL (Neurofilament Light Chain) Levels as a Predictive Marker for Long-Term Outcome After Ischemic Stroke. Stroke 2019;50:3077–84. https://doi.org/10.1161/STROKEAHA.119.026410.

[29] Wang N, Wang D, Zhou H, Xu C, Hu X, Qian Z, et al. Serum Neuropeptide Y Level is Associated with Post-Ischemic Stroke Epilepsy. Journal of Stroke and Cerebrovascular Diseases 2021;30. https://doi.org/10.1016/j.jstrokecerebrovasdis.2020.105475.

[30] Rodríguez-Castro E, Hervella P, López-Dequidt I, Arias-Rivas S, Santamaría-Cadavid M, López-Loureiro I, et al. NT-pro-BNP: A novel predictor of stroke risk after transient ischemic attack. International Journal of Cardiology 2020;298:93–7. https://doi.org/10.1016/j.ijcard.2019.06.056.

[31] Lee J, Mun S, Park A, Kim D, Lee Y-J, Kim H-J, et al. Proteomics Reveals Plasma Biomarkers for Ischemic Stroke Related to the Coagulation Cascade. J Mol Neurosci 2020;70:1321–31. https://doi.org/10.1007/s12031-020-01545-4.

[32] Zhu Y, Zhang J, Liu L, Han Y, Ge X, Zhao S. Evaluation of serum retinol-binding protein-4 levels as a biomarker of poor short-term prognosis in ischemic stroke. Bioscience Reports 2018;38:BSR20180786. https://doi.org/10.1042/BSR20180786.

[33] Abraira L, Santamarina E, Cazorla S, Bustamante A, Quintana M, Toledo M, et al. Blood biomarkers predictive of epilepsy after an acute stroke event. Epilepsia 2020;61:2244–53. https://doi.org/10.1111/epi.16648.

[34] Pawelczyk M, Glabiński A, Kaczorowska B, Baj Z. sP- and sE-selectin in stroke patients with metabolic disorders. Neurologia i Neurochirurgia Polska 2018;52:599–605. https://doi.org/10.1016/j.pjnns.2018.08.004.

[35] Gunnarsson M, Malmeström C, Axelsson M, Sundström P, Dahle C, Vrethem M, et al. Axonal damage in relapsing multiple sclerosis is markedly reduced by natalizumab. Ann Neurol 2011;69:83–9. https://doi.org/10.1002/ana.22247.

[36] Cantó E, Tintoré M, Villar LM, Costa C, Nurtdinov R, Álvarez-Cermeño JC, et al. Chitinase 3-like 1: prognostic biomarker in clinically isolated syndromes. Brain 2015;138:918–31. https://doi.org/10.1093/brain/awv017.

[37] Comabella M, Fernández M, Martin R, Rivera-Vallvé S, Borrás E, Chiva C, et al. Cerebrospinal fluid chitinase 3-like 1 levels are associated with conversion to multiple sclerosis. Brain 2010;133:1082–93. https://doi.org/10.1093/brain/awq035.

[38] Brettschneider J, Czerwoniak A, Senel M, Fang L, Kassubek J, Pinkhardt E, et al. The Chemokine CXCL13 Is a Prognostic Marker in Clinically Isolated Syndrome (CIS). PLOS ONE 2010;5:e11986. https://doi.org/10.1371/journal.pone.0011986.

[39] Högel H, Rissanen E, Barro C, Matilainen M, Nylund M, Kuhle J, et al. Serum glial fibrillary acidic protein correlates with multiple sclerosis disease severity. Mult Scler 2020;26:210–9. https://doi.org/10.1177/1352458518819380.

[40] Kassubek R, Gorges M, Schocke M, Hagenston VAM, Huss A, Ludolph AC, et al. GFAP in early multiple sclerosis: A biomarker for inflammation. Neurosci Lett 2017;657:166–70. https://doi.org/10.1016/j.neulet.2017.07.050.

[41] Andersson M, Alvarez-Cermeño J, Bernardi G, Cogato I, Fredman P, Frederiksen J, et al. Cerebrospinal fluid in the diagnosis of multiple sclerosis: a consensus report. J Neurol Neurosurg Psychiatry 1994;57:897–902.

[42] Senel M, Tumani H, Lauda F, Presslauer S, Mojib-Yezdani R, Otto M, et al. Cerebrospinal Fluid Immunoglobulin Kappa Light Chain in Clinically Isolated Syndrome and Multiple Sclerosis. PLOS ONE 2014;9:e88680. https://doi.org/10.1371/journal.pone.0088680.

[43] Chen Y-C, Yang X, Miao L, Liu Z-G, Li W, Zhao Z-X, et al. Serum level of interleukin-6 in Chinese patients with multiple sclerosis. J Neuroimmunol 2012;249:109–11. https://doi.org/10.1016/j.jneuroim.2012.04.015.

[44] Castellazzi M, Bellini T, Trentini A, Delbue S, Elia F, Gastaldi M, et al. Serum Gelatinases Levels in Multiple Sclerosis Patients during 21 Months of Natalizumab Therapy. Dis Markers 2016;2016:8434209. https://doi.org/10.1155/2016/8434209.

[45] Fissolo N, Pignolet B, Matute-Blanch C, Triviño JC, Miró B, Mota M, et al. Matrix metalloproteinase 9 is decreased in natalizumab-treated multiple sclerosis patients at risk for progressive multifocal leukoencephalopathy. Ann Neurol 2017;82:186–95. https://doi.org/10.1002/ana.24987.

[46] Galboiz Y, Shapiro S, Lahat N, Rawashdeh H, Miller A. Matrix metalloproteinases and their tissue inhibitors as markers of disease subtype and response to interferon-beta therapy in relapsing and secondary-progressive multiple sclerosis patients. Ann Neurol 2001;50:443–51. https://doi.org/10.1002/ana.1218.

[47] Vennegoor A, Rispens T, Strijbis EM, Seewann A, Uitdehaag BM, Balk LJ, et al. Clinical relevance of serum natalizumab concentration and anti-natalizumab antibodies in multiple sclerosis. Mult Scler 2013;19:593–600. https://doi.org/10.1177/1352458512460604.

[48] Ioannides ZA, Csurhes PA, Swayne A, Foubert P, Aftab BT, Pender MP. Correlations between macrophage/microglial activation marker sTREM-2 and measures of T-cell activation, neuroaxonal damage and disease severity in multiple sclerosis. Mult Scler J Exp Transl Clin 2021;7:20552173211019772. https://doi.org/10.1177/20552173211019772.
