# Supplementary material for: A unified classification approach rating clinical utility of protein biomarkers across neurologic diseases
Source: eBioMedicine. 2023 Feb 4;89:104456. doi: 10.1016/j.ebiom.2023.104456 (PMC9931915; doi:10.1016/j.ebiom.2023.104456)
Supplement: Supplementary Fig. S1 [file mmc2.pdf]

Treatment approach „Conservative“	„Progressive“	CIViC	ESMO	NCT	JCR	EDRN
		**A:** Proven/consensus association in human medicine<br>**B:** Clinical trial or other primary patient data supports association<br>**C:** Individual case reports from clinical journals	**I A:** Prospective randomized trial<br>**I B:** Prospective non-randomized trial<br>**I C:** Basket trials and trials across disease types<br>**II A:** Retrospective studies<br>**II B:** Prospective trials; endpoints not currently available	**m1 A:** Same entity: Prospective study<br>**m1 B:** Same entity: Retrospective study<br>**m1 C:** Same entity: Case reports<br>**m2 A:** Different entity: Prospective study	**Tier 1 A:** FDA approved therapy/ professional guidelines<br>**Tier 1 B:** Well-powered studies with expert consensus	**Phase 5:** Disease burden reduction studies<br>**Phase 4:** Prospective diagnostic accuracy studies<br>**Phase 3:** Retrospective studies
		**D:** In vivo or in vitro models support association<br>**E:** Indirect evidence	**III B:** Alteration with predicted impact in same pathway as I<br>**IV A:** in vivo or in vitro evidence<br>**IV B:** in silico evidence<br>**V:** Objective response but not improved outcome	**m2 B:** Different entity: Retrospective study<br>**m2 C:** Different entity: Case reports<br>**m3:** in vitro / in vivo results<br>**m4:** Biological rationale: no clinical/preclinical data available	**Tier 2 C:** FDA approval in different entity/ inclusion in clinical trial / multiple small studies with some consensus<br>**Tier 2 D:** Preclinical trials / case reports	**Phase 2:** Clinical assay development<br>**Phase 1:** preclinical exploratory studies
			**X:** Benign variants		**Tier 3:** No convincing evidence of cancer association<br>**Tier 4:** No existing evidence of cancer association	
